# Supplementary material for: Recombination and Pol ζ Rescue Defective DNA Replication upon Impaired CMG Helicase—Pol ε Interaction
Source: Int J Mol Sci. 2020 Dec 13;21(24):9484. doi: 10.3390/ijms21249484 (PMC7762974; doi:10.3390/ijms21249484)
Supplement: Supplementary file 1 [file ijms-21-09484-s001.pdf]

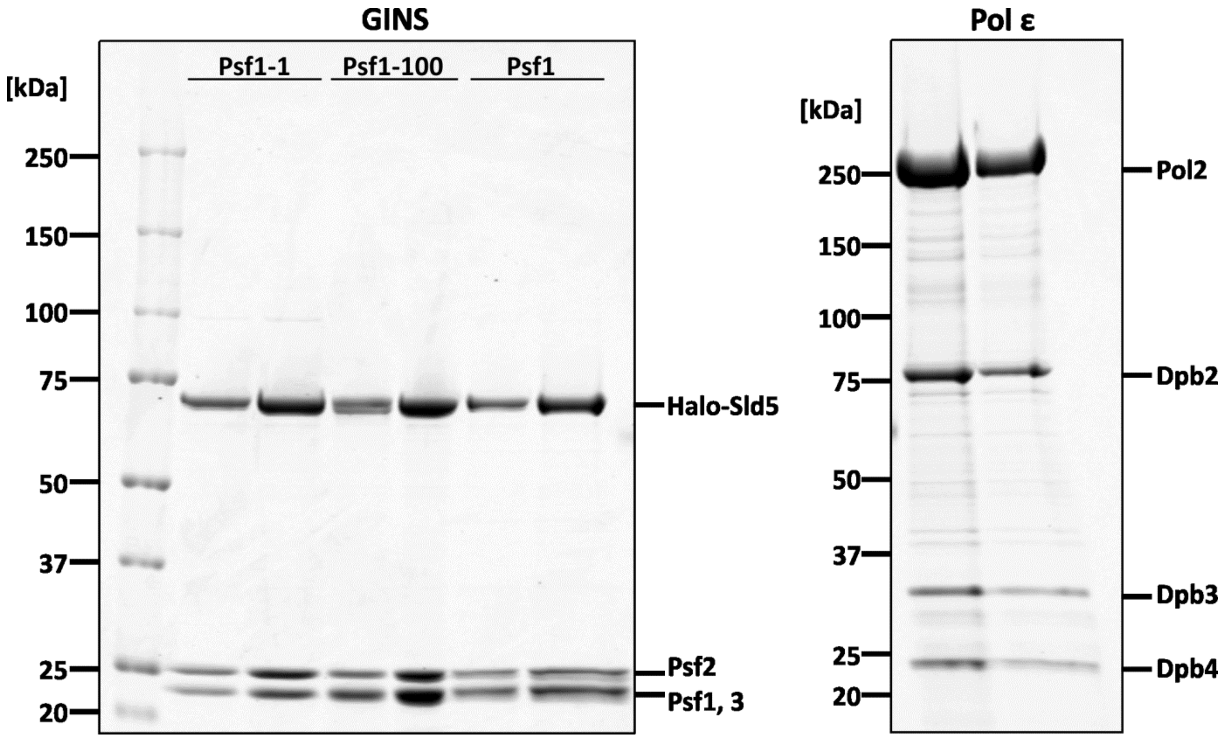

Figure S1. Gel patterns of GINS and Pol ε complex proteins used in the GINS-Pol ε *in vitro* interaction assay shown in Figure 1A.

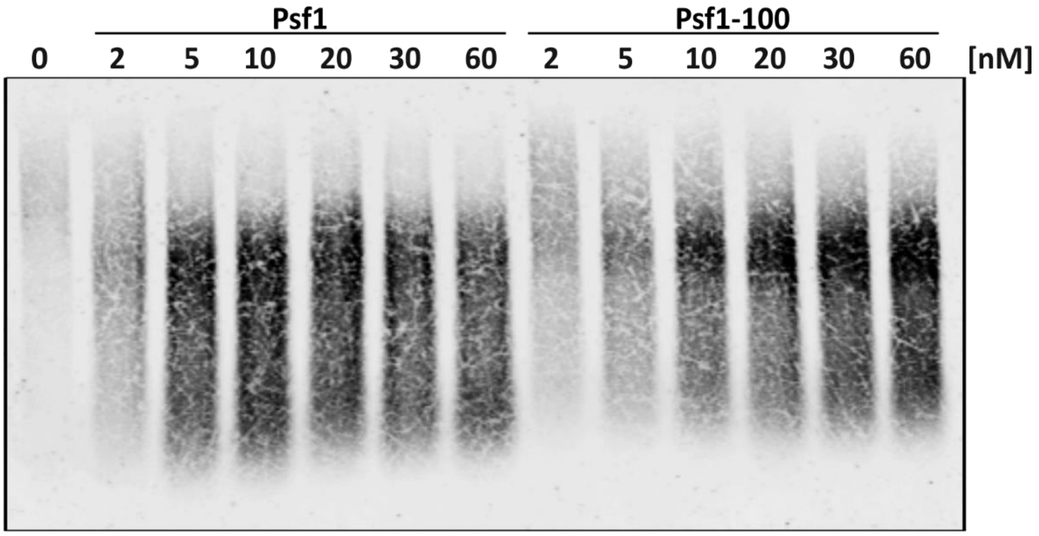

Figure S2. Detection of incorporated dNTPs in the *in vitro* replication assay shown in Figure 1B.

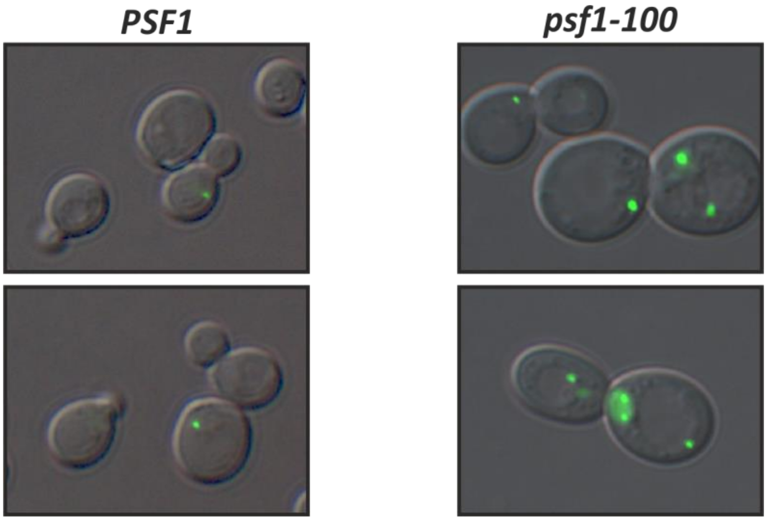

Figure S3. Representative images of *PSF1* and *psf1-100* cells with Rfa1-YFP foci.

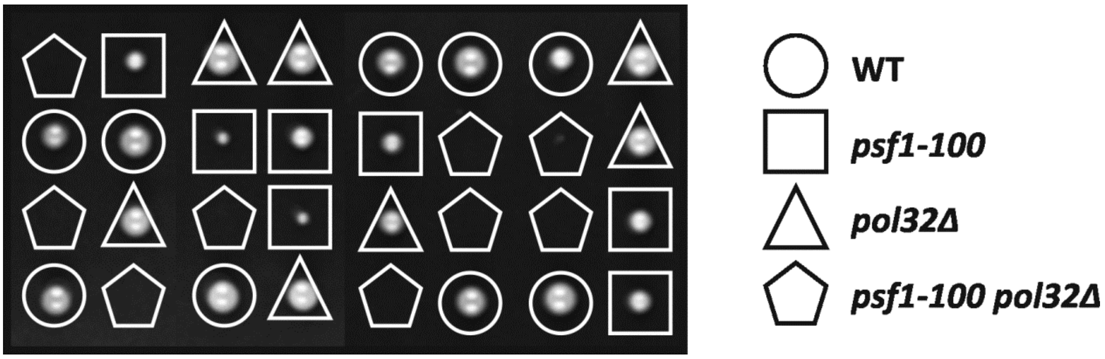

Figure S4. Synthetic lethality of the *psf1-100* mutation and *POL32* deletion. Dissection of tetrads from the *psf1-100/PSF1 pol32Δ/POL32* strain.

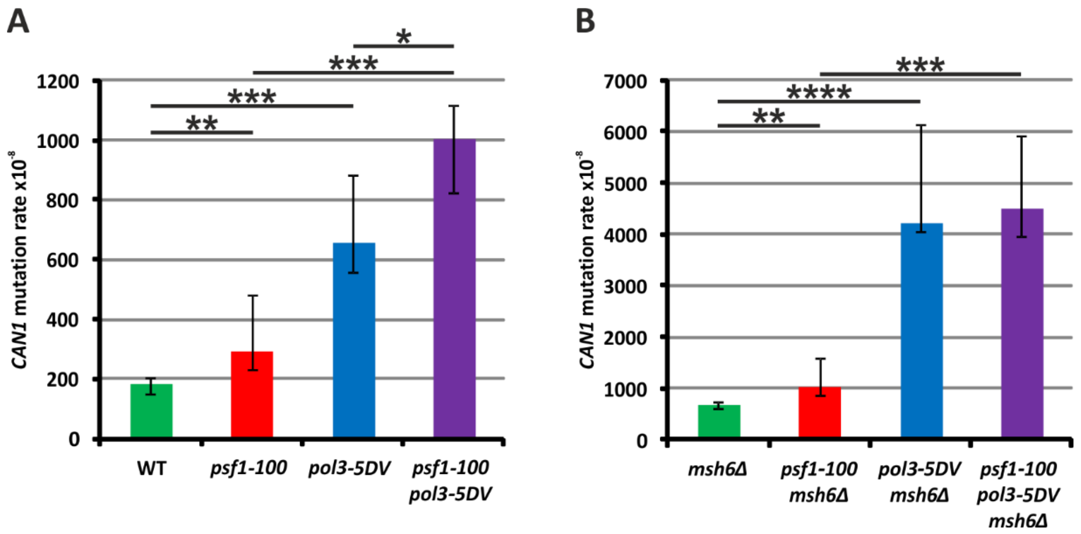

Figure S5. Spontaneous mutation rates measured in the *psf1-100 pol3-5DV* strains. The analysis was made in *MSH6* **(A)** and *msh6Δ* **(B)** backgrounds. The presented values are medians with 95% confidence intervals calculated from at least ten independent cultures. Mann-Whitney U test was used to determine the *p*-value ≤0.0001 (****); ≤0.001 (***); ≤0.01 (**); ≤0.05 (*). Exact *p*-values are shown in Supporting Table S2. Strains were constructed as follows:

The *pol3-5DV* cassette (Eco52I-linearized pY19 plasmid, kindly provided by D. Gordenin) was integrated into the *POL3* locus of the SC765 and Y1000 strains to generate Y1037 and Y1038, respectively (Table S10). Ura^+ transformants were selected and toothpicked twice onto 5-FOA plates at 30°C and verified by sequencing of a DNA fragment PCR-amplified using primers pol3-1 and pol3-2 (Table S11). Strains Y999 and Y1039 carrying deletion of the *MSH6* gene were constructed based on the SC765 or Y1000 strain, respectively by replacing the coding region of the *MSH6* gene with a DNA cassette containing the *HPH* gene, which was PCR-amplified with the primers MSH6_UPTEF and MSH6_DNTEF listed in Table S11 using pAG32 [1] as a template. Deletion of the *MSH6* open reading frame was confirmed by PCR using primers msh6UP2, msh6UO and HPH UO (Table S11). Strains Y1041, Y1045 and Y1047 were constructed by tetrad dissection from diploid strains by crossing SC778 with Y1038, Y1039 with Y1037 and Y1039 with Y1041, respectively (Table S10). *MSH6* disruption was confirmed by PCR using primers msh6UP2 and msh6UO (Table S11). The presence of *POL3* or the *pol3-5DV* allele was verified as described above. The presence of the (*psf1-100, CaURA3*) allele was confirmed by DNA sequencing of a PCR fragment amplified using primers: Inprom and dwPSF1 (Table S11). Additionally, the presence of the *psf1-100* allele was verified by a temperature sensitivity test: *psf1-100* strain does not grow at 18°C. The (*PSF1, CaURA3*) [2] was integrated into the *PSF1* locus of the Y999, Y1037 and Y1045 strains to obtain strains Y1048, Y1049 and Y1046, respectively (Table S10). The (*psf1-100, CaURA3*) cassettes [2] was integrated into the *PSF1* locus of the Y999 strain to obtain Y1044. The presence of the (*PSF1, CaURA3*) and (*psf1-100, CaURA3*) alleles was confirmed as described above.

Table S1. Statistical analysis of Rfa1, Rad51, and Rad52 foci in *psf1-100* cells presented in Figure 2A-C[1].

Figure 2A Rfa1 foci	0 foci	1 foci	2 foci	3 foci	*Row totals*
PSF1	660 (487.50) [61.04]	516 (567.57) [4.68]	81 (153.50) [34.25]	25 (73.43) [31.95]	1282
psf1-100	442 (614.50) [48.43]	767 (715.43) [3.72]	266 (193.50) [27.17]	141 (92.57) [25.34]	1616
Column totals	1102	1283	347	166	2898 (grand total)

The χ2 statistic is 236.5692. The p-value is < 0.00001. The result is significant at p < 0.05.

Figure 2B Rad51 foci	0 foci	1 foci	Row totals
PSF1	5429 (5399.90) [0.16]	9 (38.10) [22.23]	5438
psf1-100	3499 (3528.10) [0.24]	54 (24.90) [34.02]	3553
Column totals	8928	63	8991 (grand total)

The χ2 statistic is 56.6504. The *p*-value is < 0.00001. The result is significant at *p* < 0.05.

Figure 2C Rad52 foci	0 foci	1 foci	Row totals
PSF1	3106 (3056.33) [0.81]	35 (84.67) [29.14]	3141
psf1-100	4619 (4668.67) [0.53]	179 (129.33) [19.07]	4798
Column totals	7725	214	7939 (grand total)

The χ2 statistic is 49.5449. The *p*-value is < 0.00001. The result is significant at *p* < 0.05.

[1] Contingency table and χ2 test was used to determine *p*-values

Table S2. *p*-values associated with data presented in Figure S5[1].

psf1-100	*pol3-5DV*	*psf1-100 pol3-5DV*	Figure S5A
0.0017453	0.0006364	0.0001210	**WT**
	0.0034150	0.0002419	***psf1-100***
		0.0310448	***pol3-5DV***

psf1-100 msh6Δ	*pol3-5DV msh6Δ*	*psf1-100 pol3-5DV msh6Δ*	Figure S5B
0.0014510	0.0000374	0.0000483	***msh6Δ***
	0.0000197	0.0006841	***psf1-100 msh6Δ***
		0.6117559	***pol3-5DV msh6Δ***

[1] Mann–Whitney U test was used to determine *p*-values.

Table S3. *p*-values associated with data presented in Figure 2F and G[1].

psf1-100	*rad51Δ*	*psf1-100 rad51Δ*	Figure 2F
0.0017230	0.000177000	0.000176866	**WT**
	0.000007000	0.000000300	***psf1-100***
		0.000000002	***rad51Δ***

psf1-100	*mms2Δ*	*psf1-100 mms2Δ*	*pif1Δ*	*psf1-100 pif1Δ*	Figure 2G
0.0003858	0.0000030	0.0000001	0.0000016	0.00000014	**WT**
	0.0109250	0.0002505	0.0007828	0.00004534	***psf1-100***
		0.0006611	X	X	***mms2Δ***
			X	X	***psf1-100 mms2Δ***
				0.00012170	***pif1Δ***

[1] Mann–Whitney U test was used to determine *p*-values.

Table S4. *p*-values associated with data presented in Figure 3B[1].

	UV 5		UV 15	
	WT	*psf1-100*	WT	*psf1-100*
rev3	**0.00000000000102**	X	**0.00000000000051**	X
G2-REV3	0.81053233151601	X	0.20953104506541	X
psf1-100 rev3	x	0.09267807	X	**0.002038145**
psf1-100 G2-REV3	x	0.10514368	X	0.590307505

[1] Statistical T-test was used to determine *p*-values. Statistically significant differences between strains are indicated by **bold face**

Table S5. *p*-values associated with data presented in Figure 3C[1].

	UV 0		UV 5		UV 15	
	WT	*psf1-100*	WT	*psf1-100*	WT	*psf1-100*
rev3	**0.000032**	X	**0.000011**	X	**0.000011**	X
G2-REV3	0.063509	X	0.050035	X	0.768134	X
psf1-100 rev3	X	**0.000032**	X	**0.000076**	X	**0.000032**
psf1-100 G2-REV3	X	0.128305	X	0.125673	X	0.723155

[1] Mann–Whitney U test was used to determine *p*-values. Statistically significant differences between strains are indicated by **bold face**

Table S6. *p*-values associated with data presented in Figure 4A.

Viable cells (propidium iodide staining of dead cells)[1]						
rev3Δ	***rad51Δ***	***psf1-100***	***psf1-100 rev3Δ***	***psf1-100 rad51Δ***	***psf1-100 rev3Δ rad51Δ***	
**0.0395**	0.1168	**0.0439**	**<.0001**	0.0671	**0.0046**	**WT**
			**<.0001**	**0.023**	0.3349	***psf1-100***
					0.6629	***rev3Δ rad51Δ***

Colony forming units[2]						
rev3Δ	***rad51Δ***	***psf1-100***	***psf1-100 rev3Δ***	***psf1-100 rad51Δ***	***psf1-100 rev3Δ rad51Δ***	
0.1306056	0.8913413	0.0586689	0.6834744	**0.0015049**	**0.0000478**	**WT**
			**0.0333595**	0.1824849	**0.0018775**	***psf1-100***
					**0.0000007**	***rev3Δ rad51Δ***

Colony forming units/viable cells[2]						
rev3Δ	***rad51Δ***	***psf1-100***	***psf1-100 rev3Δ***	***psf1-100 rad51Δ***	***psf1-100 rev3Δ rad51Δ***	
0.2769818	0.5122757	**0.0218567**	0.1061510	**0.0018961**	**0.0000485**	**WT**
			0.2360034	0.3802150	**0.0017477**	***psf1-100***
					**0.0000006**	***rev3Δ rad51Δ***

[1] Contingency table and $\chi 2$ test was used to determine *p*-values
[2] Statistical T-test was used to determine *p*-values.
Statistically significant differences are indicated by **bold face**

Table S7. Rates, percentage and relative rates of various types of base substitutions, insertions, deletions and complex mutations for the *psf1-100 pol2-4 rev3Δ* strain.

Type of mutations	*rev3Δ*[1]		*psf1-100 rev3Δ*[1]		*pol2-4 rev3Δ*[1]		*psf1-100 pol2-4 rev3Δ*	
**Base substitutions**	**24[2] (56.5%)[3]**	**[1][4]**	**22 (51%)**	**[0.9]**	**148 (77.5%)**	**[6.2]**	**238 (65%)**	**[9.9]**
Transitions	16 (37%)	[1]	16 (36.5%)	[1.0]	48 (25%)	[3.0]	69 (19%)	[4.3]
AT→GC	3.5 (9%)	[1]	4 (8%)	[1.1]	15 (8%)	[4.3]	5 (1%)	[1.4]
GC→AT	12.5 (28%)	[1]	12 (28.5%)	[1.0]	33 (17%)	[2.6]	64 (18%)	[5.1]
Transversions	8 (19.5%)	[1]	6 (14.5%)	[0.8]	100 (52.5%)	[12]	169 (46%)	[21.1]
AT→CG	1 (2%)	[1]	1 (2.5%)	[1.0]	4 (2%)	[4.0]	5 (1%)	[4.8]
AT→TA	1.5 (3%)	[1]	< 0.5 (< 1)	[< 1]	25 (13%)	[16]	74 (20%)	[49.2]
GC→TA	3.5 (9%)	[1]	5 (12%)	[1.4]	69 (36.5%)	[19]	88 (24%)	[25.1]
GC→CG	2 (5.5%)	[1]	< 0.5 (< 1)	[< 1]	2 (1%)	[1.0]	2 (1%)	[1.2]
**Indels**	**18.5 (43.5%)**	**[1]**	**21 (49%)**	**[1.1]**	**43 (22.5%)**	**[2.3]**	**126 (35%)**	**[6.8]**
Insertions	6.5 (15%)	[1]	6 (14%)	[0.9]	13 (7%)	[2.0]	86 (24%)	[13.2]
+1	3 (7.5%)	[1]	2.5 (6%)	[0.8]	13 (7%)	[4.3]	86 (24%)	[28.5]
+2	< 0.5 (< 1)		0.5 (1%)		< 2 (< 1)		< 0.5 (< 1)	
≥+ 3	3 (7.5%)		3 (7%)		< 2 (< 1)		< 0.5 (< 1)	
Deletions	12 (28%)	[1]	15 (35%)	[1.3]	30 (15.5%)	[2.5]	40 (11%)	[3.4]
-1	7 (16%)	[1]	9 (21%)	[1.3]	18 (9%)	[2.6]	33 (9%)	[4.8]
-2	2 (4.5%)		3.5 (8%)		10 (5.5%)		7 (2%)	
≥ -3	3 (7.5%)		2.5 (6%)		2 (1%)		< 0.5 (< 1)	
**Complex[5]**	**< 0.5 (< 1)**	**[1]**	**< 0.5 (< 1)**		**< 2 (< 1)**		**< 0.5 (< 1)**	
**Total**	**42.5 (100%)**	**[1]**	**43 (100%)**	**[1.0]**	**191 (100%)**	**[4.5]**	**364 (100%)**	**[8.6]**

[1] Data for isogenic *rev3Δ*, *psf1-100 rev3Δ* and *pol2-4 rev3Δ* were previously shown [2].

[2] Rates [Can^R ($x10^{-8}$)] for particular types of mutations were calculated according to the equation MRi = (Mi/MT) x MR, where Mi is the number of mutations of a particular type, MT is the total number of mutations, and MR is the overall rate of Can^R mutations in the strain determined by fluctuation analysis (for reference see [2]). Associated *p*-values are shown in Table S8.

[3] Percentage of events for specific classes of mutations are shown in brackets.

[4] The relative rate presents the increase in mutability [the rate of mutagenesis of a particular type in a given strain is divided by the rate of mutagenesis in *rev3Δ*].

[5] Complex mutations are defined as multiple changes within short (up to 6 nt) DNA stretches.

Table S8. *p*-values associated with data presented in Figure 5 and Table S7[1].

Spontaneous Can^R mutagenesis			
psf1-100 rev3Δ	***pol2-4 rev3Δ***	***psf1-100 rev3Δ pol2-4***	
0.4479946282	**0.0000004150**	**0.0000000023**	***rev3Δ***
	**0.0000015383**	**0.0000000296**	***psf1-100 rev3Δ***
		**0.0139249353**	***pol2-4 rev3Δ***

Base substitution			
psf1-100 rev3Δ	***pol2-4 rev3Δ***	***psf1-100 rev3Δ pol2-4***	
0.1806811	**0.0000282**	**0.0000046**	***rev3Δ***
	**0.0002256**	**0.0000228**	***psf1-100 rev3Δ***
		0.1165457	***pol2-4 rev3Δ***

Insertions/deletions (indels)			
psf1-100 rev3Δ	***pol2-4 rev3Δ***	***psf1-100 rev3Δ pol2-4***	
0.732726	**0.022053**	**0.000029**	***rev3Δ***
	0.076283	**0.000588**	***psf1-100 rev3Δ***
		**0.035932**	***pol2-4 rev3Δ***

[1] contingency table and the χ2 test were used to determine *p*-values

Table S9. Statistical analysis of results presented in Figure 6[1].

Plasmid	Repeat tract	Allele	Rate x 10^{-6}	95% CI	*p*-value
**pKK2**	**Random sequence**	***PSF1***	0.77	0.70 - 1.00	**0.000000000004**
		psf1-100	2.40	2.22 - 2.80	
**pMD28**	**$(G)_{18}$**	***PSF1***	9.30	8.19 - 10.94	**0.000000001972**
		psf1-100	28.00	27.30 - 39.20	
**p51GT**	**$(GT)_{25}$**	***PSF1***	8.60	8.61 - 13.18	**0.000000000003**
		psf1-100	54.00	48.73 - 68.16	
**pMD41**	**$(AACGCAATGCG)_4$**	***PSF1***	8.00	7.38 - 8.78	**0.000000000054**
		psf1-100	17.00	17.13 - 21.99	
**pEAS20**	**$(CAACGCAATGCGTTGGATCT)_3$**	***PSF1***	86.00	76.73 - 96.39	**0.000000000011**
		psf1-100	370.00	360.20 - 607.20	

[1] Mann–Whitney U test was used to determine *p*-values

Table S10. Yeast strains used in this study.

Strain	Genotype/description	Source
	**Parent strains**	
SC765[a]	*MATα CAN1 his7-2 leu2Δ::hisG ura3Δ trp1-289 ade2-1 lys2ΔGG2899-2900*	[2]
Y1000[a]	*MATα CAN1 his7-2 leu2Δ::hisG ura3Δ trp1-289 ade2-1 lys2ΔGG2899-2900*	This work
MAB1[b]	*MATα can1-100 V9229::HPH V261553::HIS3*	[3]
MAB4 [b]	*MATa can1Δ::SUP4-o V9229::kanMX V261553::LEU2*	[3]
	**Counting of Rfa1 foci**	
Y1025	*As SC765, but* ***(RFA1-YFP, LEU2) (PSF1, CaURA3)***	This work
Y1026	*As SC765, but* ***(RFA1-YFP, LEU2) (psf1-100, CaURA3)***	This work
	**Counting of Rad52 foci**	
Y1027	*As SC765, but* ***(PSF1, CaURA3) [pWJ1344]***	This work
Y1028	*As SC765, but* ***(psf1-100, CaURA3) [pWJ1344]***	This work
	**Counting of Rad51 foci**	
Y1029	*As SC765, but* ***(PSF1, CaURA3) [pSFP119]***	This work
Y1030	*As SC765, but* ***(psf1-100, CaURA3) [pSFP119]***	This work
	**Construction of diploid strains**	
Y1001	As Y1000, but ***rad52::HPH***	This work
Y1002	As Y1000, but ***rad51::HPH***	This work
Y1003	As Y1000, but ***mms2::HPH***	This work
Y1004	As Y1000, but ***pif1::HPH***	This work
Y1005	As Y1000, but ***pol32::HPH***	This work
SC778	*As SC765, but* ***(psf1-100, CaURA3)***	[2]
SC803	*MATa CAN1 his7-2 leu2Δ::kanMX4 ura3Δ trp1-289 ade2-1 lys2ΔGG2899-2900* ***(psf1-100, LEU2)***	[2]
Y1006	*As SC765, but* ***rev3::NAT1 (psf1-100, LEU2)***	This work
Y1037	*As SC765, but* ***pol3-5DV***	This work
Y1038	*As Y1000, but* ***pol3-5DV***	This work
Y1039	*As Y1000, but* ***msh6::HPH***	This work
Y1041	*As SC765, but* ***pol3-5DV (psf1-100, CaURA3)***	This work
	**Diploid strains**	
Y1007	*MATa/α CAN1/CAN1 his7-2/his7-2 leu2Δ::hisG/leu2Δ::hisG ura3Δ/ura3Δ trp1-289/trp1-289 ade2-1/ade2-1 lys2ΔGG2899-2900/lys2ΔGG2899-2900* ***rad52::HPH/RAD52 (psf1-100, CaURA3)/PSF1*** Cross of SC778 and Y1001	This work
Y1008	*MATa/α CAN1/CAN1 his7-2/his7-2 leu2Δ::hisG/leu2Δ::kanMX4 ura3Δ/ura3Δ trp1-289/trp1-289 ade2-1/ade2-1 lys2ΔGG2899-2900/lys2ΔGG2899-2900* ***rad51::HPH/RAD51 (psf1-100, LEU2)/PSF1*** Cross of SC803 and Y1002	This work
Y1009	*MATa/α CAN1/CAN1 his7-2/his7-2 leu2Δ::hisG/leu2Δ::hisG ura3Δ/ura3Δ trp1-289/trp1-289 ade2-1/ade2-1 lys2ΔGG2899-2900/lys2ΔGG2899-2900* ***rad51::HPH/RAD51 rev3::NAT1/REV3 (psf1-100, LEU2)/PSF1*** Cross of Y1006 and Y1002	This work
Y1010	*MATa/α CAN1/CAN1 his7-2/his7-2 leu2Δ::hisG/leu2Δ::kanMX4 ura3Δ/ura3Δ trp1-289/trp1-289 ade2-1/ade2-1 lys2ΔGG2899-2900/lys2ΔGG2899-2900* ***mms2::HPH/MMS2 (psf1-100, LEU2)/PSF1*** Cross of SC803 and Y1003	This work
Y1011	*MATa/α CAN1/CAN1 his7-2/his7-2 leu2Δ::hisG/leu2Δ::kanMX4 ura3Δ/ura3Δ trp1-289/trp1-289 ade2-1/ade2-1 lys2ΔGG2899-2900/lys2ΔGG2899-2900* ***pif1::HPH/PIF1 (psf1-100, LEU2)/PSF1*** Cross of SC803 and Y1004	This work
Y537	*MATa/α CAN1/CAN1 his7-2/his7-2 leu2Δ::hisG/leu2Δ::hisG ura3Δ/ura3Δ trp1-289/trp1-289 ade2-1/ade2-1 lys2ΔGG2899-2900/lys2ΔGG2899-2900* ***pol32::HPH/POL32 (psf1-100, CaURA3)/PSF1*** Cross of SC778 and Y1005	This work
Y1042	*MATa/α CAN1/CAN1 his7-2/his7-2 leu2Δ::hisG/leu2Δ::hisG ura3Δ/ura3Δ trp1-289/trp1-289 ade2-1/ade2-1 lys2ΔGG2899-2900/lys2ΔGG2899-2900* ***msh6::HPH/MSH6 pol3-5DV/POL3*** Cross of Y1039 and Y1037	This work
Y1040	*MATa/α CAN1/CAN1 his7-2/his7-2 leu2Δ::hisG/leu2Δ::hisG ura3Δ/ura3Δ trp1-289/trp1-289 ade2-1/ade2-1 lys2ΔGG2899-2900/lys2ΔGG2899-2900* ***pol3-5DV/POL3 (psf1-100, CaURA3)/PSF1*** Cross of Y1038 and SC778	This work
Y1043	*MATa/α CAN1/CAN1 his7-2/his7-2 leu2Δ::hisG/leu2Δ::hisG ura3Δ/ura3Δ trp1-289/trp1-289 ade2-1/ade2-1 lys2ΔGG2899-2900/lys2ΔGG2899-2900* ***pol3-5DV/POL3 msh6::HPH/MSH6 (psf1-100, CaURA3)/PSF1*** Cross of Y1039 and Y1041	This work

	**Measurement of spontaneous mutagenesis in *psf1-100* derivatives defective in Pol ζ, template switch or HR**	
Y1012	*As SC765, but* ***(PSF1, LEU2)***	This work
Y1013	*As SC765, but* ***(psf1-100, LEU2)***	This work
Y1014	*As SC765, but* ***rad51::HPH (PSF1, LEU2)***	This work
Y1015	*As SC765, but* ***pif1::HPH (PSF1, LEU2)***	This work
Y1016	*As SC765, but* ***mms2::HPH (PSF1, LEU2)***	This work
Y1017	*As SC765, but* ***rev3::NAT1 (PSF1, LEU2)***	This work
Y1018	*As SC765, but* ***rad51::HPH rev3::NAT1 (PSF1, LEU2)***	This work
Y1019	*As SC765, but* ***rad51::HPH (psf1-100, LEU2)***	This work
Y1020	*As SC765, but* ***rad51::HPH rev3::NAT1 (psf1-100, LEU2)***	This work
Y1021	*As SC765, but* ***pif1::HPH (psf1-100, LEU2)***	This work
Y1022	*As SC765, but* ***mms2::HPH (psf1-100, LEU2)***	This work
Y1006	*As SC765, but* ***rev3::NAT1 (psf1-100, LEU2)***	This work
	**Measurement of spontaneous mutagenesis in *psf1-100* derivatives defective in Pol δ proofreading / MMR**	
SC766	*As SC765, but* ***(PSF1, CaURA3)***	[2]
SC778	*As SC765, but* ***(psf1-100, CaURA3)***	[2]
Y999	*As SC765, but* ***msh6::HPH***	This work
Y1048	*As SC765, but* ***msh6::HPH (PSF1, CaURA3)***	This work
Y1049	*As SC765, but* ***pol3-5DV (PSF1, CaURA3)***	This work
Y1044	*As SC765, but* ***msh6::HPH (psf1-100, CaURA3)***	This work
Y1041	*As SC765, but* ***pol3-5DV (psf1-100, CaURA3)***	This work
Y1045	*As SC765, but* ***pol3-5DV msh6::HPH***	This work
Y1046	*As SC765, but* ***pol3-5DV msh6::HPH (PSF1, CaURA3)***	This work
Y1047	*As SC765, but* ***pol3-5DV msh6::HPH (psf1-100, CaURA3)***	This work
	**Analysis of recombination events**	
Y1031	*As MAB1, but* ***(PSF1, CaURA3)***	This work
Y1032	*As MAB1, but* ***(psf1-100, CaURA3)***	This work
Y1033	*As MAB4, but* ***(PSF1, CaURA3)***	This work
Y1034	*As MAB4, but* ***(psf1-100, CaURA3)***	This work
Y1035	*MATα/MATa can1-100/ can1Δ::SUP4-o V9229::HPH/ V9229::kanMX V261553::HIS3/V261553::LEU2* ***(PSF1, CaURA3/PSF1, CaURA3)*** Cross of Y1031 and Y1033	This work
Y1036	*MATα/MATa can1-100/ can1Δ::SUP4-o V9229::HPH/ V9229::kanMX V261553::HIS3/V261553::LEU2* ***(psf1-100, CaURA3/psf1-100, CaURA3)*** Cross of Y1032 and Y1034	This work
	**Measurement of spontaneous and induced mutagenesis in *psf1-100* cells with *G2-REV3***	
SC766	*As SC765, but* ***(PSF1, CaURA3)***	[2]
SC778	*As SC765, but* ***(psf1-100, CaURA3)***	[2]
Y514	*As SC765, but* ***rev3::LEU2 (PSF1, CaURA3)***	[4]
Y522	*As SC765, but* ***rev3::LEU2 (psf1-100, CaURA3)***	[4]
Y1023	*As SC765, but* ***(G2-REV3, natNT2) (PSF1, CaURA3)***	This work
Y1024	*As SC765, but* ***(G2-REV3, natNT2) (psf1-100, CaURA3)***	This work
	**Analyzis of the *CAN1* mutation spectrum**	
SC665	*MAT**a** CAN1 his7-2 leu2Δ::kanMX4 ura3Δ trp1-289 ade2-1 lys2ΔGG2899-2900* ***rev3::LEU2*** **(*PSF1, CaURA3*)**	[2]
SC808	*MAT**a** CAN1 his7-2 leu2Δ::kanMX4 ura3Δ trp1-289 ade2-1 lys2ΔGG2899-2900* ***rev3::LEU2*** **(*psf1-100, CaURA3*)**	[2]
SC658	*MAT**a** CAN1 his7-2 leu2Δ::kanMX4 ura3Δ trp1-289 ade2-1 lys2ΔGG2899-2900* ***pol2-4 rev3::LEU2*** **(*PSF1, CaURA3*)**	[2]
SC660	*MAT**a** CAN1 his7-2 leu2Δ::kanMX4 ura3Δ trp1-289 ade2-1 lys2ΔGG2899-2900* ***pol2-4 rev3::LEU2*** **(*psf1-100, CaURA3*)**	[2]
	**Measurement of repeated DNA tracts instability in *psf1-100***	
SC801	*MAT**a** CAN1 his7-2 leu2Δ::kanMX4 ura3Δ trp1-289 ade2-1 lys2ΔGG2899-2900* ***(PSF1, LEU2)***	[2]
SC803	*MAT**a** CAN1 his7-2 leu2Δ::kanMX4 ura3Δ trp1-289 ade2-1 lys2ΔGG2899-2900* ***(psf1-100, LEU2)***	[2]

[a] This strain is a derivative of ΔI(-2)I-7B-YUNI300 [5]

[b] This strain is a derivative of W303 [3]

Table S11. Primers used in this study.

Primer name	Sequence 5'→3'/application	
	**PCR amplification and DNA sequencing of *CAN1* locus**	
MGCANFF	AAGAGTGGTTGCGAACAGAG	
MGCANRR	GGAGCAAGATTGTTGTGGTG	
Can_1666	ATATTTGACAGGGAACAAGT	
Can_1963	GATGGCTCTTGGAACGGA	
Can_2241	TGTCAAGGACCACCAAAG	
Can_2465	GTAACTCGTCACGAGAGA	
	**Gene disruptions**	
REV3_UPTEF	CAATACAAAACTACAAGTTGTGGCGAAATAAAATGTTTGGAAATGAGATCTGTTTAGCTTGCC	
REV3_DNTEF	ATAACTACTCATCATTTTGCGAGACATATCTGTGTCTAGATTATTCGAGCTCGTTTTCGACAC	
RAD52_UPTEF	ACGAAAAATATAGCGGCGGGCGGGTTACGCGACCGGTATCGAATGGAGATCTGTTTAGCTTGCC	
RAD52_DNTEF	ATAATGATGCAAATTTTTTATTTGTTTCGGCCAGGAAGCGTTTCAATTCGAGCTCGTTTTCGACAC	
RAD51_UPTEF	ACGTAGTTATTTGTTAAAGGCCTACTAATTTGTTATCGTCATATGGAGATCTGTTTAGCTTGCC	
RAD51_DNTEF	AAGTAAACCTGTGTAAATAAATAGAGACAAGAGACCAAATACCTAATTCGAGCTCGTTTTCGACAC	
MMS2_UPTEF	ATTCTGTATATGCAACGTAGAAGAAAGCAGCGTTTACACAAAAATGAGATCTGTTTAGCTTGCC	
MMS2_DNTEF	TGGCTTGGAATGCTGCAAATACTGTTTAGGAAAAAGTAGATAACTATTCGAGCTCGTTTTCGACAC	
PIF1_UPTEF	TTATCCATTGAGCGATTAGCTTACTTGTATCAATCAATTTTACATGAGATCTGTTTAGCTTGCC	
PIF1_DNTEF	ATAGCAGTTTGTATTCTATATAACTATGTGTATTAATATGTACTTATTCGAGCTCGTTTTCGACAC	
POL32_UPTEF	ATAATATTTCACATTAACTAACAACCAGAAATAGGCTTTAGTTAACTCAATCGGTAATTA	
POL32_DNTEF	CATTTGTATTATACATTACATCACAATTAGTAATGGAAAGTGTTTGGAAAAAAAAGAAG	
MSH6_UPTEF	CAGATAAGATTTTTTAATTGGAGCAACTAGTTAATTTTGACAAAGCCAATTTGAACTCCAAAAGATCTGTTTAGCTTGCC	
MSH6_DNTEF	CAACGACCAAAACTTTAAAAAAAATAAGTAAAAATCTTACATACATCGTAAATGAAAATATTCGAGCTCGTTTTCGACAC	
	**Verification of gene disruptions**	
HPH UO	ACAGACGTCGCGGTGAGTTCAG	
HPH DO	TCGCCGATAGTGGAAACCGACG	
NAT1 UO	ACCGGTAAGCCGTGTCGTCAAG	
NAT1 DO	GCTTCGTGGTCGTCTCGTACTC	
msh6UP2	GAATCCTTGGAGGAAGAC	
msh6UO	TAAAGTCGCTGGAGTAGG	
REV3 A	AATTCTGCCAATCTATTTGATCTTG	
REV3 B	TCTGATTTAGAGGATGATCTAACCG	
REV3 C	TAAATGAAGACCATAGAGCAGAACC	
REV3 D	CACCAGATAGAGTTTTGAACGAAAT	
RAD52 A	GATTCAACAACTCCCTTGGCGTC	
RAD52 B	CAACCTTCGATGTATGCAATCCTG	
RAD52 C	CGCGTGAAACCACACCAA	
RAD52 D	TACGACACATGGAGGAAAGAAAAAC	
RAD51 A	CCAATCTAGTTTAGCTATCCTGCAA	
RAD51 B	AAAGTGTGACATAGCTGGGACTTAC	
RAD51 C	GTAAGTCCCAGCTATGTCACACTTT	
RAD51 D	AATTTTTCTCTTCACTCCCCTAAAA	
PIF1 A	AAAGGCGCGTCTTAATTTTCTTCACT	*Saccharomyces cerevisiae* Genome Deletion Project
PIF1 B	GTGCGATACGTTTTTGAGTAAAAGAAA	
PIF1 C	ATCAAGTTCATTCATTGGTTTCCGAC	
PIF1 D	CTTTTTTCTATCGAAGGAGGTTCACC	
MMS2 A	CACCACTATTGCTCATTTTGTACTG	
MMS2 B	TAATATCGTCGCTATCAGCTAAACC	
MMS2 C	AAGATAAATCTACCATGCGTCAATC	
MMS2 D	TATTTATTATTGGCTTGGACTGGAG	
POL32 A	AATTCTCGATCAGTATGCCTCAATA	
POL32 B	TTTGTCTAGAGGTTTCCTTGTCATC	
	**PCR amplification and DNA sequencing of *PSF1* or *psf1-100* locus**	
InProm	AGCTAGGTTCCAAGAAGGCT	
dwPSF1	CCAGCTTGAAAGCATCGATA	
	**PCR amplification and DNA sequencing of *pol2-4* locus**	
pol2-1	CAGTGGGTCGTACATCTC	
pol2-4_1	ATCAGTTATTCGAGGCCAGG	

	**PCR amplification and DNA sequencing of *pol3-5DV* locus**
pol3-1	GAGTCTGTGTTCTCTTCG
pol3-2	CCATTAGGTGTTATGACG
	**Construction of the *G2-REV3, natNT2* fusion cassette**
S1_REV3	GTATTTGAGTCAATACAAAACTACAAGTTGTGGCGAAATAAAATGTTTGGAAATGCGTACGCTGCAGGTCGAC
S4_REV3	ATTTAGAGGATGATCTAACCGTATCGCTCTGTATTGTGTCGTTCGACTCCCTCGACATCGATGAATTCTCTGTCG
	**PCR amplification and DNA sequencing of *G2-REV3, natNT2* locus**
Rev3 A	AATTCTGCCAATCTATTTGATCTTG
Rev3-R3	TGACCACTCACATGGCGCTTTG
Rev3up	GATAAGTATTCACTAACACC
Rev3_R1	CTTTCACCGTGCGATGGGTC
prCLB2	TCGCTCGTTTGTCAGAAG
	**Confirmation of the *RFA1-YFP* fusion cassette**
RFA6231R	ACGGTTCACAATCCCTACAG
RFA7367F	GCCGCAACGCAAACTTCATC
YFP9451R	CTTCGGGCATGGCACTCTTG

REFERENCES

1. Goldstein, A.L.; McCusker, J.H. Three new dominant drug resistance cassettes for gene disruption in Saccharomyces cerevisiae. *Yeast* **1999**, *15*, 1541–1553, doi:10.1002/(SICI)1097-0061(199910)15:14<1541::AID-YEA476>3.0.CO;2-K.
2. Grabowska, E.; Wronska, U.; Denkiewicz, M.; Jaszczur, M.; Respondek, A.; Alabrudzinska, M.; Suski, C.; Makiela-Dzbenska, K.; Jonczyk, P.; Fijalkowska, I.J. Proper functioning of the GINS complex is important for the fidelity of DNA replication in yeast. *Mol. Microbiol.* **2014**, *92*, 659–680, doi:10.1111/mmi.12580.
3. Barbera, M.A.; Petes, T.D. Selection and analysis of spontaneous reciprocal mitotic cross-overs in Saccharomyces cerevisiae. *Proc. Natl. Acad. Sci. U. S. A.* **2006**, *103*, 12819–12824, doi:10.1073/pnas.0605778103.
4. Szwajczak, E.; Fijalkowska, I.J.; Suski, C. The CysB motif of Rev3p involved in the formation of the four-subunit DNA polymerase ζ is required for defective-replisome-induced mutagenesis. *Mol. Microbiol.* **2017**, *106*, 659–672, doi:10.1111/mmi.13846.
5. Pavlov, Y.I.; Newlon, C.S.; Kunkel, T.A. Yeast origins establish a strand bias for replicational mutagenesis. *Mol. Cell* **2002**, *10*, 207–213, doi:10.1016/S1097-2765(02)00567-1.
